# Modulation of Renal Injury by Variable Expression of Myo-Inositol Oxygenase (MIOX) via Perturbation in Metabolic Sensors

**DOI:** 10.3390/biomedicines8070217

**Published:** 2020-07-16

**Authors:** Isha Sharma, Fei Deng, Yashpal S. Kanwar

**Affiliations:** Department of Pathology, Northwestern University, Chicago, IL 60611, USA; isha.sharma1@northwestern.edu (I.S.); fei.deng@northwestern.edu (F.D.)

**Keywords:** Myo-inositol oxygenase (MIOX), mitochondrial functions, metabolic sensors

## Abstract

Obesity is associated with perturbations in cellular energy homeostasis and consequential renal injury leading to chronic renal disease (CKD). Myo-inositol oxygenase (MIOX), a tubular enzyme, alters redox balance and subsequent tubular injury in the settings of obesity. Mechanism(s) for such adverse changes remain enigmatic. Conceivably, MIOX accentuates renal injury via reducing expression/activity of metabolic sensors, which perturb mitochondrial dynamics and, if sustained, would ultimately contribute towards CKD. In this brief communication, we utilized MIOX-TG (Transgenic) and MIOX*^KO^* mice, and subjected them to high fat diet (HFD) administration. In addition, *ob/ob* and *ob*/MIOX*^KO^* mice of comparable age were used. Mice fed with HFD had increased MIOX expression and remarkable derangements in tubular injury biomarkers. Decreased expression of p-AMPKα (phospho AMP-activated protein kinase) in the tubules was also observed, and it was accentuated in MIOX-TG mice. Interestingly, *ob/ob* mice also had decreased p-AMPKα expression, which was restored in *ob*/MIOX*^KO^* mice. Parallel changes were observed in Sirt1/Sirt3 (silent mating type information regulation 2 homolog), and expression of other metabolic sensors, i.e., PGC-1α (Peroxisome proliferator-activated receptor gamma coactivator 1-alpha) and Yin Yang (YY-1). In vitro experiments with tubular cells subjected to palmitate-BSA and MIOX-siRNA had results in conformity with the in vivo observations. These findings link the biology of metabolic sensors to MIOX expression in impaired cellular energy homeostasis with exacerbation/amelioration of renal injury.

## 1. Introduction

Worldwide epidemic prevalence of obesity is well-known, especially in developed countries. According to the estimates of World Health Organization (WHO), by the year 2025 obesity will affect 18% of men and 21% of women worldwide, meaning that health related maladies affecting various organ systems will become major issues necessitating immediate attention and rigorous scientific investigations (http://s3-eu-west-1.amazonaws.com/wof-files/WOF_Missing_the_2025_Global_Targets_Report_FINAL_WEB.pdf). In this regard, obesity has emerged as one of the major risk factors for the development of chronic renal disease in recent years [1,2]. Compounding it are other associated factors including hypertension and diabetes, which contribute to the progression of renal injury resulting in the acceleration of chronic kidney disease (CKD) and leading to an end stage renal disorder (ESRD) [3,4,5]. Nevertheless, obesity can be regarded as an independent risk factor in the progression of CKD since it is believed to be associated with glomerular hyperfiltration, aberrant expression of certain adipokines, e.g., leptin and adiponectin, insulin resistance, activation of the renin-angiotensin-aldosterone system (RAAS), increased cellular oxidant stress and synthesis of molecules involved in the process of fibrosis, i.e., extracellular matrix (ECM) proteins [5,6].

In terms of ECM pathobiology of the kidney, the glomerular compartment has drawn major attention in various investigations of obesity related disorders and, traditionally, glomerulosclerosis or glomerular fibrosis is regarded as one of the hallmarks of CKD [7,8]. The tubulo-interstitial compartment, although less well studied, also possibly undergoes similar pathobiological changes in the state of obesity and these changes may include metabolic cellular disturbances in the tubules followed by tubular atrophy and interstitial fibrosis, ultimately contributing to CKD [9,10,11,12]. Conceivably, the onset of such tubular changes ensues cellular perturbations, especially in the proximal tubules. The latter are enriched with mitochondria and thus are readily amenable to metabolic perturbations, and are regarded as the major site of the energy sensing compartment of the kidney [13,14]. For instance, they are at the forefront to handle the large amounts of glucose filtered across the glomerulus and thus are subjected to various stresses, the most important being oxidant stress, since mitochondria are one of major sites for the generation of reactive oxygen species (ROS) [15]. In terms of energy sensing properties, besides handling of glucose by the tubules, they also respond to high fat diet (HFD) administration or states of obesity, where free fatty acids have been described to induce renal injury both in vitro and in vivo [9,10]. Administration of a HFD is also known to adversely influence the expression of some metabolic sensors including deacetylating enzymes, i.e., sirtuins, and one of the important metabolic enzymes, i.e., AMP-activated protein kinase (AMPK) [9,16,17,18].

Normally, AMPK phosphorylates some metabolic enzymes and GTP binding proteins and in doing so activates and stimulates fatty acid oxidation and glucose uptake, and thus plays a role in maintaining energy balance in various cells [19,20]. Incidentally, it also regulates an important transcriptional coactivator, known as peroxisome proliferator-activator receptor (PPAR-γ) coactivator (PGC-1α), a master regulator of mitochondrial biogenesis [21,22]. The expression/activity of AMPK is reduced in some renal pathological states, which ultimately progress to CKD [23]. In addition to AMPK, another set of molecules relevant to energy balance, and other cellular processes, include sirtuins [18,24,25]. They are NAD^+^-dependent deacetylating enzymes and modulate the activity of other enzymes. Some are expressed in mitochondria and are involved in the biology of metabolism, fuel sensing and insulin resistance [25,26]. Sirtuins’ targets include some transcription coactivators like PGC-1α, and transcription factors, e.g., Ying Yang-1 (YY-1) [27,28,29,30]. The latter is a vital regulator of oxidative phosphorylation (OXPHOS) [29,31]. Overall, the biology of AMPK, sirtuins, PGC-1α, and YY-1 are interwoven and conceivably could regulate the functionality of renal proximal tubules enriched with mitochondria.

Myo-inositol oxygenase (MIOX) is another essential metabolic enzyme that is highly expressed in proximal tubules, which also to a large extent modulates the biology of the renal tubular compartment [32]. It catalyzes Myo-inositol in the glucuronate-xylulose (GX) pathway. Previous studies suggested that MIOX upregulation in diabetic or acute tubular injury states leads to redox imbalance and induction of oxidant stress, apoptosis and acceleration of TGF-β (Transforming growth factor beta) signaling, ultimately progressing to renal fibrosis [33,34,35]. Besides altered redox balance, it is conceivable that MIOX upregulation may potentially perturb cellular energy sensors in the mitochondrial-rich proximal tubules, and thus may contribute towards renal injury in the settings of obesity. In view of the above discussion, we initiated studies to assess the status of metabolic sensors in mice with up- or down-regulation of MIOX following administration of a high fat diet (HFD), while also addressing an important key question, that is, if there is an exacerbation or amelioration of renal tubular injury.

## 2. Materials and Methods

### 2.1. Antibodies and Reagents

HK-2 cells, a human proximal tubular cell line, were purchased from the American Type Culture Collection (ATCC). Other reagents were purchased from the following vendors, and their catalogue numbers are included within brackets. Abcam (Cambridge, MA, USA): anti-SIRT1 (ab12193), -PGC-1α (ab54481); Cell Signaling Technology(Danvers, MA, USA): anti-phospho-AMPKα (2535); Origene Technologies (Rockville, MD, USA): siRNA universal control (SICOO1) MIOX siRNA (SR310776); Life Technologies: TO-PRO-3-iodide (T3605), Fast SYBR Green Mix (4367659); Sigma (St. Louis, MO, USA): Dulbecco’s Modified Eagle’s Medium (DMEM), anti-β-actin (A5441); Thermo Fischer Scientific (Waltham, MA, USA): LipofectamineTM 2000 Transfection Reagent (11668027), anti-SIRT3 (HPA026809), -YY1 (SAB4200302); Research Diet Inc (New Brunswick, NJ, USA): High fat diet chow (HFD) (5.24 kcal/g, 60% fat, 20% protein, 20% carbohydrate, D12492). Secondary antibodies were purchased from Sigma as follows: antirabbit IgG-horseradish peroxidase (HRP; A0545), mouse IgG-HRP (A9917), rabbit IgG-FITC (F9887). Palmitate BSA (P-BSA) was prepared as described earlier [10]. The MIOX antibody is available in our laboratory and its preparation has been described previously [32].

### 2.2. Generation of MIOX Transgenic, Knock Out and Double Mutant Mice

MIOX-TG (MIOX transgenic) and MIOX-KO (MIOX knockout) mice have been generated in our lab as previously reported [33,34,35]. To generate the double mutant for MIOX^-/-^ and B6.Cg-Lep*^ob^*/J (Stock number: 000632), mutant MIOX*^KO^* male mice were mated with heterozygous *ob/ob* female mice. This mating resulted in double heterozygous male and females, and their intercross breeding generated double mutants for MIOX^-/-^ (MIOX*^KO^*) and *ob/ob*, i.e., *ob*/MIOX*^KO^*. The genetic validity of the double mutant mice was confirmed by polymerase chain reaction (PCR) analyses.

### 2.3. Animals Studies

Wild type (WT) C57BL/6J, MIOX-TG and MIOX*^KO^* male mice were fed a high fat diet (HFD) starting at the age of 8 weeks. The animals were acclimatized for one week in rooms with 12 h light/dark cycle with a constant temperature of 22 °C and 50% humidity. They had access to water and food ad libitum. The three strains of mice were divided into two different groups (*n* = 6). The mice were fed an HFD or normal chow for four months and then were sacrificed. Likewise, 8 weeks old *ob/ob* and *ob*/MIOX*^KO^* mice (*n* = 6) were kept under similar ambience. They were then fed normal chow only and sacrificed after 4 months. At the time of sacrifice, kidneys samples were collected for various studies. The kidney cortices were dissected out, and were used for various morphological and biochemical studies. All animal procedures used in this study were approved (2018-2043) by the Animal Care and Use Committee of Northwestern University on 8 July 2018.

### 2.4. Cell Culture Experiments

Initially, HK-2 cells were grown in a keratinocyte serum-free media (Life Technologies) in the presence of 5 ng/mL recombinant EGF (Epidermal growth factor), 0.05 mg/mL bovine pituitary extract and penicillin-streptomycin solution (100 U/mL penicillin and 100 µg/mL streptomycin). Subsequently, the HK-2 cells were grown on collagen-coated dishes and maintained in DMEM, containing 5 mM D-glucose, 10% FBS (Foetal Bovine Serum) and penicillin streptomycin solution in an atmosphere of 5% CO_2_ at 37 °C. The MIOX overexpressing cell line was generated in our laboratory as described previously [34,35]. The general strategy used for cell culture experiment was as follows: ~2 × 10^5^ cells were seeded in 55 cm^2^ culture dishes and maintained to achieve 80% confluency. Following trypsinization, the cells (~1 × 10^5^) were plated on 2.2 cm^2^ coverslips in DMEM medium containing 2% FBS for morphological studies. Cells were then treated with palmitate bovine serum albumin (P-BSA, 100 μM) for 24 h. BSA was used as a control. For gene disruption studies, the cells were grown in the presence of 50 μM MIOX-siRNA, and scrambled siRNA was used as a control.

### 2.5. RNA Isolation and Real-Time PCR

TRIzol (Invitrogen (Waltham, MA, USA)) reagent was used for extraction of total RNA isolation from kidney cortices. Go Script reverse transcription system (Promega (Madison, WI, USA)) was used for cDNA synthesis. The synthesized cDNA was used to quantify the mRNA levels of various genes using Step One Plus System Real Time PCR (Applied Biosystems, Foster City, CA, USA). The PCR reaction mixture included 1 µg of cDNA, 50 nmol/L sense and antisense primers and 1× FAST SYBRGreen (a total of 10 µL). For amplifying target and internal control regions, the reaction conditions used were as follows: 94 °C for 2 min, followed by 39 cycles of 94 °C for 20 s each, 60 °C for 15 s, 72 °C for 15 s and the final extension cycle of 4 min at 72 °C. β-actin was used as an internal control for normalization of gene expression, and the relative abundance of mRNA of each gene was calculated and expressed as fold change. The single peak in melt curve indicated generation of a single PCR product during amplification. The primers used were as follows: MIOX: forward: 5′-TGTCTTCACCACCTACAAGCTC-3′, reverse: 5′-GGCCTC CATGACTGTCATTTTC-3′; Kidney Injury Molecule-1 (KIM-1): forward: 5′-GGAAGTAAAGGGGGTAGTGGG-3′, reverse: 5′-AAGCAGAAGATGGGCATTGC-3′; Neutrophil gelatinase-associated lipocalin (NGAL): forward: 5′-GCCCAGGACTCA ACTCAGAA-3′, reverse: 5′-GACCAGGATGGAGGTGACAT-3′; β-actin: forward, 5′-GGTCATCACCATTGGCAATGAG-3′, reverse 5′-TACAGGTCTTTGCGGATGTCC-3′.

### 2.6. Immunofluorescence Microscopy

HK-2 cells were seeded (0.5 × 10^5^) on 2.2 cm^2^ cover slips. The cells were either subjected to BSA, P-BSA or concomitantly treated with MIOX siRNA for 24 h, following which the cells were washed with PBS for two times 5 min each. The cells were then allowed to fix with 4% formaldehyde in PBS at 22 °C for 15 min. They were then washed three times with ice cold PBS for 5 min and then permeabilized with 0.25% triton X-100 in PBS for 10 min at 22 °C. PBS-T containing 2% BSA was used for blocking and the cells were blocked for 30 min at 22 °C. The cells were then incubated with primary antibody (YY-1) using the same buffer for 15 h at 4 °C. The cells were washed two times with 1X PBS for 5 min each. They were then incubated with FITC (Fluorescein isothiocyanate)-conjugated secondary antibody in 2% BSA for 1 h at 22 °C. The cells were rewashed two times with PBS. The coverslips with attached cells were inverted and mounted onto glass slides with one drop of DAKO mounting medium. The expression of p-AMPKα was also assessed on cryostat sections of snap-frozen kidney tissues. Briefly, 4-µm cryostat sections were prepared from OCT (Optimal cutting temperature)-embedded tissues. Sections were air-dried and then washed with PBS. These sections were then fixed in 10% phosphate buffered formalin for 20 min. Sections were washed with PBS and then permeabilized with 0.25% Triton-X100 in PBS for 10 min and then washed twice with PBS for 5 min at 22 °C. They were immersed in PBST containing 2% BSA for 30 min at 22 °C, following which they were incubated with primary antibody (p-AMPKα) for 15 h at 4 °C. They were incubated with secondary antibody and stained with TO-PRO-3 iodide for 15 min to visualize the nuclei. The tissue sections were examined using a UV microscope equipped with epi-illumination.

### 2.7. Immunoblot Analysis of Relevant Proteins

Kidney tissues harvested from various strains of mice were diced into 1 mm^3^ fragments and then homogenized in RIPA (Radioimmunoprecipitation assay) buffer containing protease-phosphatase inhibitor cocktail at 4 °C for 30 min. Likewise, HK-2 cells were subjected to P-BSA treatment while others were concomitantly treated with MIOX siRNA. Cells were scraped from the culture dishes, pelleted and lysed in RIPA buffer. Following vortexing, they were centrifuged at 12,000× *g* at 4 °C for 10 min. The supernatant was collected in a clean centrifuge tube and the protein concentration was estimated by the Bradford method. Lysates were stored at −70 °C to be used for various experiments. Various samples with equal amounts of protein (30 µg) were subjected to 10–12% SDS-PAGE (Sodium Dodecyl Sulfate–Polyacrylamide Gel Electrophoresis). The fractionated proteins were then transferred to PVDF (polyvinylidene difluoride) membranes by electroblotting. The membrane blots were immersed in 5% nonfat dry milk in TBS-T solution containing Tween-20. The blots were then incubated with various primary antibodies for 12–15 h at 4 °C in 2% nonfat dry milk in TBST (1x Tris-Buffered Saline, 0.1% Tween^®^ 20). Blots were washed with TBST three times and incubated with secondary antirabbit or antimouse antibodies. Autoradiograms were prepared using an Enhanced ChemiLuminence (ECL) Kit (Amersham Bioscience, Waltham, MA, USA). β-actin was used as a loading control.

### 2.8. Statistics

Statistical analyses were carried out using GraphPad Prism (version 7.01). Significance was determined using one-way ANOVA with Dunn’s multiple comparisons. Results were expressed as mean ± SD of 4–6 samples in each variable.

## 3. Results

### 3.1. High Fat Diet Administration Leads to Increased Expression of Myo-Inositol Oxygenase (MIOX) and Perturbations in the Biomarkers of Renal Injury in Different Strains of Mice

**Figure 1:**
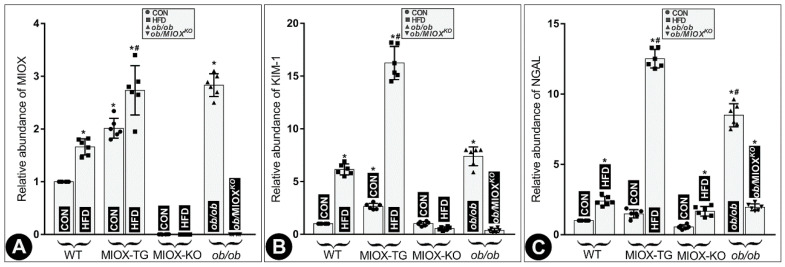
High fat diet (HFD) administration leads to increased expression of Myo-inositol oxygenase (MIOX) and perturbations in the biomarkers of renal injury in different strains of mice. Panel (**A**): following HFD administration, an increase in the MIOX expression was observed in wild type (WT) mice and a much higher increase was seen in MIOX transgenic (MIOX-TG) mice. No increase in MIOX expression was observed in MIOX*^KO^* mice fed with HFD. The *ob/ob* mice had very high MIOX expression and it was reduced to undetectable levels in *ob*/MIOX*^KO^* mutant mice. Panel (**B**,**C**): a parallel increase in the levels of both the renal injury markers, i.e., Kidney Injury Molecule-1 (KIM-1) and neutrophil gelatinase-associated lipocalin (NGAL) RNA, was observed in various strains of mice following HFD administration, and the most notable increase was observed in MIOX-TG mice. No increase in their expression was observed in MIOX*^KO^* mice. The *ob/ob* mice had a very high expression of KIM-1 and NGAL and it was remarkably reduced in *ob*/MIOX*^KO^* mice. (*n* = 6; * *p* < 0.05 compared with the control group, # *p* < 0.05 compared with the HFD group, one-way ANOVA with Dunn’s multiple comparisons).

RNA isolated from kidneys of different strains of mice was subjected to real-time PCR. The results suggested an increase in MIOX expression in WT mice following a high fat diet (HFD) treatment. MIOX-TG mice fed with HFD showed much higher increase in MIOX expression compared to control (CON) mice fed normal chow (Figure 1A). No expression of MIOX was observed in MIOX*^KO^* mice and no discernible increase was observed in mice fed with HFD (Figure 1A). Like MIOX-TG mice the *ob/ob* mice had a remarkably high MIOX expression, which was reduced to almost undetectable levels in MIOX*^KO^* mice crossbred with *ob/ob* (*ob*/MIOX*^KO^*) (Figure 1A). Increased MIOX expression was associated with a proportionate deterioration in renal functions. Two well-studied biomarkers relevant to renal tubular injury include Kidney Injury Molecule-1 (KIM-1) and neutrophil gelatinase-associated lipocalin (NGAL), and expression of both the markers are known to increase in states of renal injury [36,37,38]. There was a parallel increase in the abundance of both the KIM-1 and NGAL RNA in the renal cortices of various strains of mice along with increased expression of MIOX following HFD administration. The most notable increase was observed in MIOX-TG mice, suggesting remarkable worsening of renal injury (Figure 1B,C). A minimum, or no, increase in their expression was observed in MIOX*^KO^* mice. Interestingly, the *ob/ob* mice had a notably high expression of KIM and NGAL, and levels of both were remarkably reduced in *ob*/MIOX*^KO^* mice (Figure 1B,C), suggesting that over-expression of MIOX is associated with increased levels of renal injury biomarkers.

### 3.2. MIOX Overexpression Alters the Expression of the Metabolic Sensor p-AMPKα in Renal Proximal Tubules in State of Obesity

**Figure 2:**
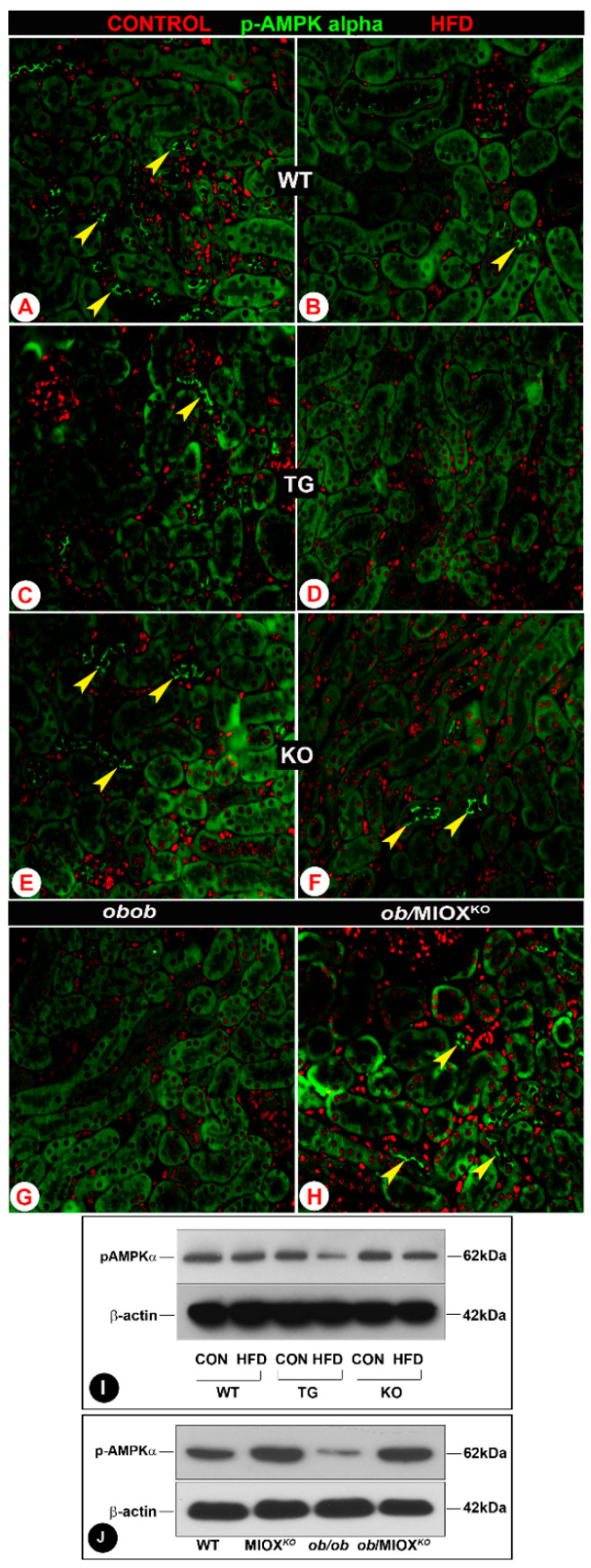
MIOX overexpression alters the expression of the metabolic sensor p-AMPKα in renal proximal tubules in a state of obesity. Panels (**A**–**H**): by immunofluorescence microscopy, a decrease in the expression p-AMPKα in the proximal tubular epithelia (arrowheads) was observed in WT, MIOX-TG and MIOX-KO mice following HFD administration. The lowest expression was observed in MIOX-TG mice, and MIOX-KO mice had discernible expression comparable to WT mice. The *ob/ob* mice had no expression of p-AMPKα, while it became detectable in some of the renal tubular epithelia in *ob*/MIOX*^KO^* mice (magnification 200×). Panels (**I**,**J**): Western blot data revealed changes in the expression of p-AMPKα that were parallel to those observed by immunofluorescence microscopy in various strains of mice in a state of obesity.

AMPKα is known to restore energy balance during metabolic stress and it is regarded as one of the major modulators during a state of diabetes or obesity [9,12,16]. Decrease in the expression of activated AMPKα has been observed in some renal disease states [12]. In view of this, renal expression of p-AMPKα was assessed in various strains of mice. The kidneys of wild type mice (WT) had discernible expression of p-AMPKα in the proximal tubular epithelia as assessed by immunofluorescence microscopy (Figure 2A). The expression was especially confined to the apical surface of the brush border epithelium of renal tubules. With the administration of HFD, a significant decrease in the expression p-AMPKα was observed in the renal epithelial apical surface (Figure 2B). The mice with MIOX overexpression had minimal p-AMPKα expression in the renal tubules (Figure 2C). With the administration of an HFD the expression of p-AMPKα became undetectable in MIOX-TG mice (Figure 2D). MIOX*^KO^* mice had p-AMPKα expression similar to that seen in WT mice kidneys, and it decreased following HFD administration (Figure 2E,F). The *ob/ob* mice had no expression of p-AMPKα, and it became detectable in some of the renal tubular epithelia in double knockout mice, i.e., *ob*/MIOX*^KO^* (Figure 2G,H). Western blot data of kidney cortices revealed changes in the expression of p-AMPKα that were parallel to those observed by immunofluorescence microscopy (Figure 2I,J). A remarkable decrease in the expression of p-AMPKα was observed in MIOX-TG mice subjected to HFD administration. While there was no expression of p-AMPKα in kidneys of *ob/ob* mice, a notable restoration of its expression was observed in mice crossbred with MIOX*^KO^*, i.e., *ob*/MIOX*^KO^*.

### 3.3. Modulation of Expression of Sirtuins and Transcription Factors (PGC-1α and YY-1) by MIOX

**Figure 3:**
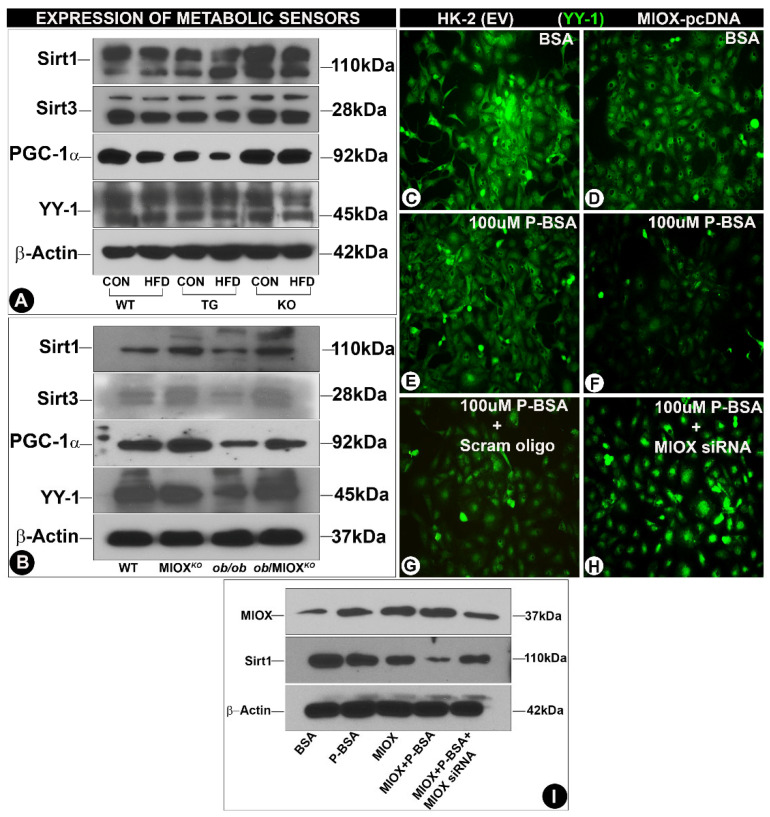
Modulation of expression of sirtuins and transcription factors (PGC-1α and YY-1) by MIOX. Panels (**A**,**B**): immunoblotting data indicated a decreased expression of Sirt1, Sirt3 and peroxisome proliferator-activated receptor gamma coactivator 1-alpha (PGC-1α) and Ying-Yang1 (YY-1) following HFD administration in various strains of mice. The most notable reduction was seen in MIOX-TG mice prior to and following HFD treatment. The ob/ob mice also had reduced expression, and it was considerably restored in ob/MIOXKO mice. Panels (**C**–**H**): immunofluorescence microscopy revealed reduced expression of YY-1 in HK-2 cells (proximal tubular cell line) transfected with MIOX pcDNA, which was further decreased following treatment with palmitate-BSA (P-BSA). The exposure of MIOX-siRNA to P-BSA-treated HK-2 cells partially restored the expression of YY-1 (magnification: 200×). Panel (**I**): Western blot data indicated the changes in the expression of MIOX and Sirt1, which basically recapitulated the changes observed in YY-1 transcription factor in HK-2 cells after various treatments.

Sirtuins (Sirts) play a pivotal role in regulating cellular homeostasis [25]. Their expression changes in pathological states that lead to perturbations in the metabolic environment [18,24]. A mild decease in the renal tubular expression of Sirt1and Sirt3 was observed in WT mice following HFD administration (Figure 3A), while a notable decrease was observed, especially that of Sirt3 in MIOX-TG mice fed with HFD. A very mild decrease in the expression of Sirt1 or Sirt3 was observed in MIOX*^KO^* mice administered with an HFD (Figure 3A). The *ob/ob* mice had reduced expression of Sirt1and Sirt3 relative to WT or MIOX*^KO^* mice (Figure 3B). Renal expression was considerably increased in *ob/ob* mice crossbred with MIOX*^KO^*, i.e., *ob*/MIOX*^KO^* (Figure 3B). The two major transcription factors that are relevant to the biology of sirtuins and energy metabolism include peroxisome proliferator-activated receptor gamma coactivator 1-alpha (PGC-1α) and Ying-Yang1 (YY-1). They are the master regulators of mitochondrial biogenesis and mitochondrial gene expression, respectively, especially in cells enriched with mitochondria, e.g., renal proximal tubules [13,14]. Like the Sirts, parallel changes were observed in the expression of PGC-1α and YY-1. A mild decrease in their expression was observed following HFD administration to WT mice (Figure 3A), while a notable decrease in their expression was observed in kidneys of MIOX-TG mice given an HFD. No discernible change was seen in the MIOX*^KO^* mice subjected to HFD treatment. Along these lines, the *ob/ob* mice had reduced expression of PGC-1α and YY-1 relative to WT or MIOX*^KO^* mice (Figure 3B). Renal expression was notably increased in the double mutant mice, i.e., *ob*/MIOX*^KO^*. These findings suggested that differential expression of MIOX had a significant influence on the levels of metabolic sensors and the genes relevant to mitochondrial biogenesis. To confirm if free fatty acids directly modulate the expression of genes related to metabolic sensors and mitochondrial biogenesis in vitro experiments were performed, where HK-2 cells, a proximal tubular cell line, were exposed to palmitate-bovine serum albumin (P-BSA). By immunoblotting procedures, increased expression of MIOX was observed following P-BSA treatment (Figure 3I, lane 2). The expression was also increased in cells transfected with MIOX-pcDNA, which was highly accentuated following concomitant exposure of P-BSA (Figure 3I, lanes 3 & 4). Interestingly, MIOX expression was notably reduced with the treatment of MIOX-siRNA (Figure 3I, lane 5). With respect to MIOX, negative correlative changes were noted in the expression of Sirt1. A decrease in the expression was observed in cells treated with P-BSA (Figure 3I, lane 2) and a drastic decrease was observed in cells transfected with MIOX-pcDNA and exposed to P-BSA (Figure 3I, lane 4). The expression of Sirt1 was partially restored following MIOX-siRNA treatment (Figure 3I, lane 5). By immunofluorescence microscopy, the HK-2 cells transfected with MIOX-pcDNA had low expression YY-1 (Figure 3D vs. Figure 3C). Treatment of 100 µM P-BSA further decreased the expression of YY-1 in both nontransfected and transfected cells (Figure 3E,F), while MIOX gene disruption led to a remarkable revival of YY-1 expression in transfected cells treated with 100 µM P-BSA (Figure 3H vs. Figure 3G), suggesting MIOX expression modulated the biology of deacetylating enzymes and transfection factors involved in energy metabolism.

## 4. Discussion

Our previous studies indicated a potential role of MIOX, an enzyme highly expressed in renal proximal tubules, in the pathogenesis of tubular injury in states of hyperglycemia or diabetes, and accentuation of renal injury following high fat diet (HFD) administration [10,32,35]. A collective message of these studies pointed towards the notion that MIOX accelerates tubular injury and, at times, tubulo-interstitial fibrosis via heightening of oxidant stress, cellular apoptosis, ferroptosis and increased de novo synthesis of extracellular matrix (ECM) proteins [34,35]. It is conceivable that MIOX up- or down-regulation could modulate the expression or activity of metabolic sensors, since the proximal tubules have very high concentration of mitochondria which apparently modulate a number of cellular energy-dependent transport processes and metabolic events [39,40,41]. Moreover, this may mean that MIOX expression could heavily influence the functionality of tubules, populated with abundant mitochondria, by perturbing some metabolic sensors, as was the subject matter of the current investigation. These sensors include the enzymes AMPKα and sirtuins, and transcription factors and co-activators YY-1 and PGC-1α.

The foremost question that needed to be addressed was if obesity or HFD administration induces acute tubular damage, which would be correlative with the up- or down-regulation of MIOX. In this regard, we utilized MIOX*^KO^* and MIOX-TG mice [33,34,35]. In addition, we crossbred MIOX-KO (MIOX*^KO^*) with leptin-deficient *ob/ob* mice to generate an *ob*/MIOX*^KO^* in order to assess if the disruption of MIOX confers a rescuing effect in states of obesity with respect to tubulo-interstitial injury reflected in the levels of tubular injury biomarkers, i.e., KIM-1 and NGAL (Figure 1).

Obesity-related chronic kidney disease (CKD) progression is well-known [1,2,3,4,5]. This progression is believed to be related to altered renal hemodynamics which adversely affect the homeostasis of the glomerulus, i.e., glomerulopathy [3,5,42,43]. Conceivable mechanism(s) proposed include increased efferent arteriolar vasoconstriction in states of obesity leading to glomerular hyperfiltration, increased activity of endothelial/mesangial cells, podocytopathy, albuminuria and, ultimately, glomerulosclerosis [5]. Apparently, in such a scenario the metabolism-related adipokines, i.e., leptin and adiponectin, modulate the outcome of renal glomerular damage. Most of the above discussion pertains to glomerular pathobiology, while sparse discussion has been devoted to renal proximal tubular epithelial injury in state of obesity.

Conceivably, the proximal tubular epithelia may also be affected in obesity-related disorders, like diabetes [9,10,38,39]. In fact, they may be more adversely affected being enriched in mitochondria, which are the sites of energy-dependent processes and thus respond readily to metabolic perturbations [14,39,40,41]. It is worth mentioning that adiponectin via AdipoR1 and AdipoR2 receptors may perturb the expression or activity of an important metabolic sensor, i.e., 5′-AMP-activated protein kinase (AMPK) in proximal tubular epithelia as in endothelial cells, since both cell types express these receptors [6,44]. Having these receptors on the proximal tubular epithelia, it is likely that adiponectin can affect the expression or activity, i.e., phosphorylation of AMPK and protect the target cells, conceivably by attenuating oxidant stress [6]. Conversely, in states of obesity that are associated with a tremendous degree of oxidant stress it is likely that the phosphorylation of AMPK would be reduced, as elegantly pointed out by Kumar and his colleagues [9,16]. Our studies also support this contention where we observed reduced phosphorylation of AMPK (p-AMPKα) following HFD administration (Figure 2). This notion is also further strengthened by current studies where there was remarkable attenuation of p-AMPKα in MIOX-TG mice, the strain that has been described to be associated with increased oxidant stress, as alluded to in our earlier publications (Figure 2) [33,34,35]. Increased oxidant stress in this transgenic strain of mice, i.e., MIOX-TG, would be expected to exacerbate tubular injury. Also, increased expression of tubular injury markers, i.e., kidney injury molecule (KIM-1) and neutrophil gelatinase-associated lipocalin (NGAL), following HFD administration would be in line with the anticipated results seen in the current investigation (Figure 1). Serum urea and creatinine levels were also notably high (unpublished data). As a corollary to the above adverse events, one would deduce that the MIOX*^KO^* would have reduced oxidant stress and increased p-AMPKα expression, and thus these mice would shield or protect the tubules from cellular injury in states of obesity. Indeed, reduced tubular injury and restoration of p-AMPKα expression in MIOX*^KO^* mice crossbred with *ob/ob* (*ob*/MIOX*^KO^*), as observed in this investigation, readily attests to this contention (Figure 1 and Figure 2).

One of the AMPK downstream targets described in the literature seem to be sirtuins, especially Sirt1, a cytoplasmic/nuclear sirtuin [18,45]. Sirtuins are NAD^+^-dependent protein deacetylases and are also regarded as one of the critical metabolic sensors which respond to various metabolic perturbations in target tissues like renal proximal tubular epithelia. AMPK could increase NAD^+^ availability and may phosphorylate Sirt1, thus stimulating Sirt1′s activity [30]. The role of Sirt1 in the pathobiology of glucose and lipid metabolism, while exerting its effect via endowed deacetylase activity, has been well described in the literature [18]. It is interesting to note that inhibition of AMPK downregulates Sirt1 which predisposes glomerular podocytes to undergo cellular damage, e.g., apoptosis [45]. Likewise, one may expect that down-regulation of Sirt1 would induce tubular damage in states of obesity. The fact that HFD administration to WT or MIOX-TG mice and *ob/ob* mice led to reduced expression of Sirt1 would support this assumption (Figure 3). Further ancillary support for this notion was derived from experiments in which palmitate-BSA was shown to downregulate the expression of Sirt1 (Figure 3). The noteworthy information of these experiments pertains to the fact that marked downregulation of Sirt1 was observed in MIOX-TG mice, and crossbreeding of MIOX*^KO^* mice with *ob/ob* restored the expression in *ob*/MIOX*^KO^* mice, suggesting MIOX expression modulates the activity of Sirt1 besides that of p-AMPKα. The fact that MIOX-siRNA treatment also normalized the expression of Sirt1 in in vitro experiments would suggest that MIOX is an upstream negative regulator of Sirt1. This effect may be due to MIOX upregulation, which leads to relative deficiency of NAD^+^, perturbations in NAD^+^/NADH ratio and redox imbalance, as described in the glucuronate-xylulose (G-X) pathway [35]. Like Sirt1, parallel changes were seen in the reduced expression of Sirt3 in various strains of mice subjected to HFD administration, and restoration of expression following crossbreeding of MIOX*^KO^* mice with *ob/ob*, thus rescuing them from tubular injury (Figure 3). Sirt3 is primarily expressed in the mitochondrial matrix and is expected to also serve as a pivotal metabolic sensor with respect to the biology of the renal proximal tubular compartment enriched with mitochondria.

Other metabolic sensors relevant to the pathobiology of mitochondria include transcriptional coactivator, known as peroxisome proliferator-activator receptor (PPAR-γ) coactivator (PGC-1α) that regulates mitochondrial biogenesis, and Ying Yang-1 (YY1), a transcription factor that regulates oxidative phosphorylation (OXPHOS), a process that ultimately yields ATP via the electron transport chain system. Sirt1 interacts and deacetylates PGC-1α in an NAD^+^-dependent manner and, in doing so, induces gluconeogenesis and maintains glucose homeostasis [28]. PGC-1α activation can also occur following phosphorylation by AMPK [14]. Besides carbohydrate metabolism, PGC-1α also participates in the regulation of lipid metabolism and conceivably it is down-regulated in states of obesity [46]. This is also supported by our studies, where HFD administration led to a decreased expression of PGC-1α, suggesting that down-stream targets of AMPK or Sirt1 are indeed affected (Figure 3). Like the up-stream modulators, PGC-1α was highly expressed in the MIOX*^KO^* mice and exerted a powerful rescuing effect in double mutant *ob*/MIOX*^KO^* mice, suggesting that MIOX expression has far-reaching downstream effects (Figure 3). HFD administration not only affected the genes relevant to mitochondrial biogenesis or gluconeogenesis, i.e., PGC-1α, but also those that are modulators of oxidative phosphorylation (OXPHOS) i.e., YY-1. YY-1 is a multifunctional zinc-finger transcription factor which is regulated by acetylation and deacetylation processes, and it binds to PGC-1α to control mitochondrial oxidative functions [27,29]. Like PGC-1α, the expression of YY-1 was slightly reduced following HFD administration in various strains of mice and in *ob/ob* mice, and it was restored in mutant *ob*/MIOX*^KO^* mice, which suggested a congruent role of these two molecules as an integral transcriptional complex, as suggested in earlier publications [29]. The notion of modulation of YY-1 in states of obesity is strengthened by our in vitro cell culture studies where a reduction in its expression was seen following exposure to palmitate-BSA and accentuated by concomitant transfection of MIOX-pcDNA (Figure 3). The fact that YY-1 expression was restored following treatment with MIOX-siRNA would suggest an integral role of MIOX in fatty acids-induced injury.

**Figure 4:**
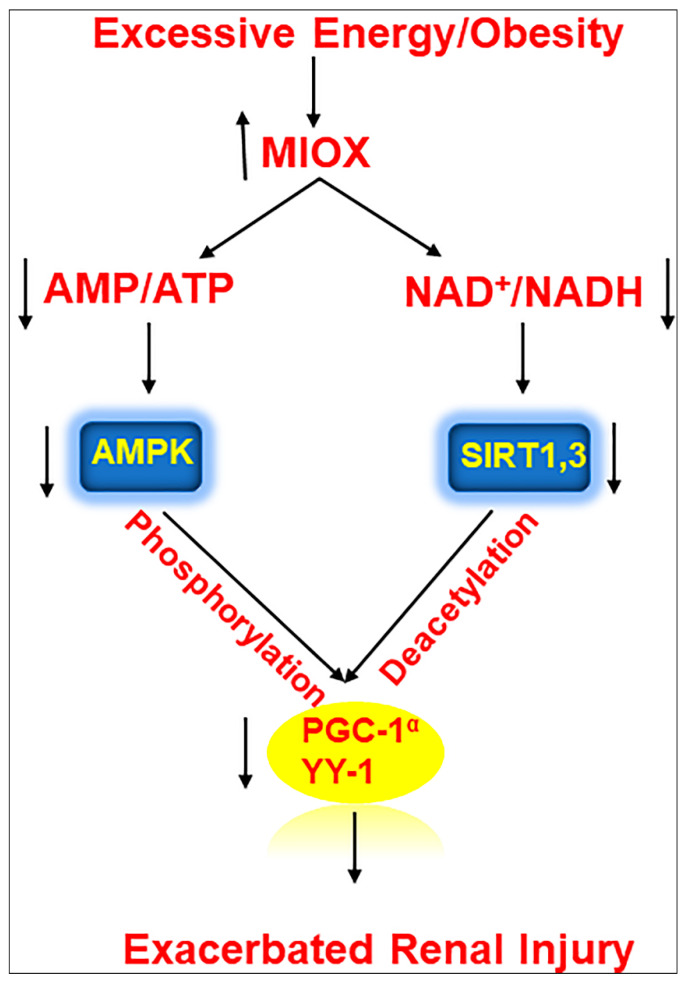
Schematic representation of series of events involved in the exacerbation of renal injury with MIOX overexpression in the setting of obesity. Hyperlipidemic ambience increases the expression of MIOX. Subsequently there are perturbations in cellular redox potential. Conceivably, this is followed by downregulation of various metabolic sensors and related modulators including p-AMPKα, Sirt1/Sirt3, PGC1-α and YY-1, ultimately leading to an accentuation of renal injury.

In summary, this investigation highlighted a pathogenetic pivotal role of a renal proximal tubular enzyme, i.e., MIOX, in modulating the expression of a series of metabolic sensors relevant to lipid metabolism, leading to an accentuation of tubulo-interstitial injury in various murine models in states of obesity (Figure 4).
