# Correction: Sharma et al. Modulation of Renal Injury by Variable Expression of Myo-Inositol Oxygenase (MIOX) via Perturbation in Metabolic Sensors. *Biomedicines* 2020, *8*, 217

**DOI:** 10.3390/biomedicines12112610

**Published:** 2024-11-15

**Authors:** Isha Sharma, Fei Deng, Yashpal S. Kanwar

**Affiliations:** Department of Pathology, Northwestern University, Chicago, IL 60611, USA; isha.sharma1@northwestern.edu (I.S.); fei.deng@northwestern.edu (F.D.)

In the original publication [1], there was a mistake in Figure 3 in the published version. Figure 3G got duplicated while assembling Figure 3. The corrected Figure 3 is shown below. The authors state that the scientific conclusions are unaffected. This correction was approved by the Academic Editor. The original publication has also been updated.

## Figures and Tables

**Figure 3:**
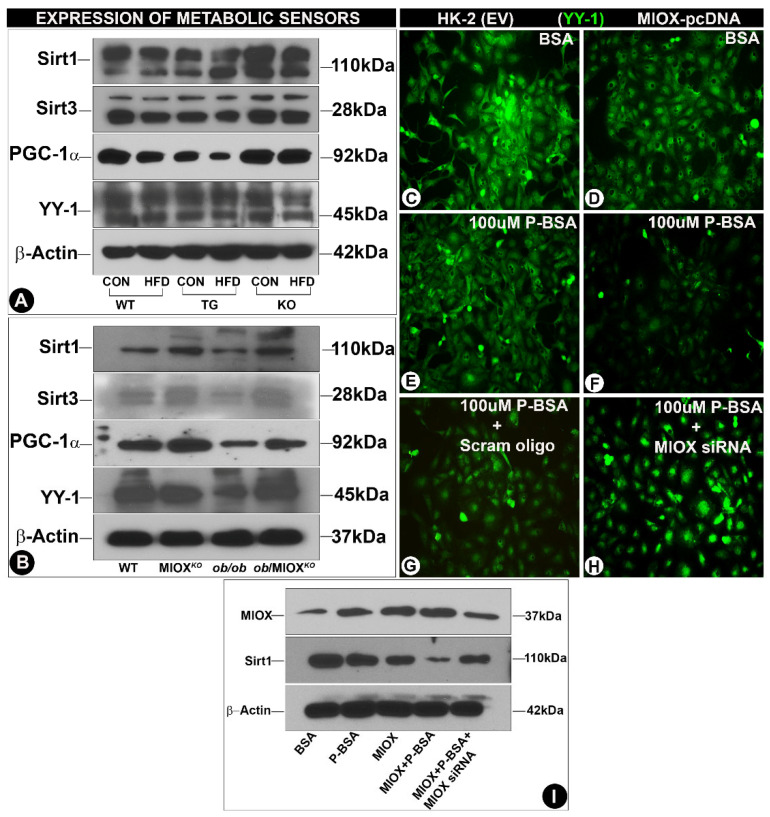
Modulation of expression of sirtuins and transcription factors (PGC-1α and YY-1) by MIOX. Panels (**A**,**B**): immunoblotting data indicated a decreased expression of Sirt1, Sirt3 and peroxisome proliferator-activated receptor gamma coactivator 1-alpha (PGC-1α) and Ying-Yang1 (YY-1) following HFD administration in various strains of mice. The most notable reduction was seen in MIOX-TG mice prior to and following HFD treatment. The ob/ob mice also had reduced expression, and it was considerably restored in ob/MIOXKO mice. Panels (**C**–**H**): immunofluorescence microscopy revealed reduced expression of YY-1 in HK-2 cells (proximal tubular cell line) transfected with MIOX pcDNA, which was further decreased following treatment with palmitate-BSA (P-BSA). The exposure of MIOX-siRNA to P-BSA-treated HK-2 cells partially restored the expression of YY-1 (magnification: 200×). Panel (**I**): Western blot data indicated the changes in the expression of MIOX and Sirt1, which basically recapitulated the changes observed in YY-1 transcription factor in HK-2 cells after various treatments.
